# Dynamic optimization of high-bandwidth multi-receiver signals for civil aircraft flight tests telemetry

**DOI:** 10.1371/journal.pone.0341948

**Published:** 2026-03-24

**Authors:** Tianchang Liu, Chen Zhao

**Affiliations:** 1 School of Mechanical and Electronic Engineering, Wuhan University of Technology, Wuhan, China; 2 COMAC Shanghai Aircraft Flight Test Co., Ltd., Shanghai, China; University of Lagos Faculty of Engineering, NIGERIA

## Abstract

Civil aircraft flight tests are distinguished by parallel multi-task operations, multi-sensor collaboration, and high-frequency data acquisition. The exponential growth in telemetry data generated in a single flight imposes stringent requirements on the high-bandwidth transmission capabilities of telemetry systems and the real-time optimization of multi-receiver signals. This study focuses on addressing the dynamic optimization of high-bandwidth multi-receiver signals during flight tests, aiming to enhance demodulation accuracy and ensure stable real-time transmission of large-scale telemetry data. A channel model for complex scenarios was constructed to analyze signal redundancy and dynamic switching in multi-receiver links, while an improved routing protocol integrated with multi-dimensional signal evaluation was investigated. A modular simulation model for the multi-receiver telemetry system was developed within a highly dynamic wireless ad hoc network environment. By enhancing the AODV and LEACH routing protocols, multi-path backup and energy consumption optimization were achieved. A multi-dimensional signal optimization algorithm was utilized to dynamically evaluate and fuse bit synchronization and frame synchronization via voting, enabling real-time selection of the optimal received signal in complex environments. The findings indicate that high-bandwidth multi-receiver signal optimization and enhanced routing strategies effectively alleviate challenges associated with multipath fading, channel obstruction, and high-speed mobility in flight test scenarios, thereby facilitating real-time channel monitoring and dynamic switching while improving data timeliness and accuracy. Simulation results demonstrate that under high-dynamic conditions, the proposed algorithm reduces the average BER to 3e^-6^ at 10 dB SNR, improves the frame synchronization success rate to 98.9%, and reduces invalid channel switching frequency by approximately 84% compared to traditional methods.

## 1. Introduction

Civil aircraft flight testing is a critical phase for verifying aircraft performance and safety. As the core transmission carrier for flight test data, the reliability of the telemetry system directly affects the integrity, real-time performance, and validity of the data. With the explosive growth in the volume of civil aircraft flight test data, dynamic optimization of high-bandwidth, multi-receiver signals have become a research hotspot in telemetry systems.

Despite advancements in telemetry technologies, several critical challenges remain. First, the conflict between high data bandwidth requirements and limited communication resources is becoming more prominent, and the real-time performance of existing algorithms in high-bandwidth scenarios is often insufficient. Second, there is a lack of efficient solutions for signal redundancy, conflicts, interference suppression, and resource allocation in multi-receiver links. Third, in actual flight environments with highly variable conditions, both the real-time transmission performance and robustness of traditional methods struggle to meet the transmission demands of high-bandwidth, multi-receiver signals. Specifically, synchronization stability in dynamic environments urgently needs improvement. Therefore, how to achieve dynamic adaptation in complex multi-channel, multi-receiver scenarios has become the main bottleneck restricting high-precision, low-latency transmission of flight test data.

This study focuses on addressing the dynamic optimization of high-bandwidth, multi-receiver signals during flight tests. The primary research objectives are: (1) to establish channel models for complex scenarios such as mountainous areas and high speeds condition; (2) to adopt a dynamic signal optimization mechanism based on the fusion of multiple indicators such as signal strength and bit error rate. This mechanism aims to achieve optimal signal selection in multi-point reception scenarios through real-time evaluation, ensuring the consistency and stability of data transmission. The value of this research lies in breaking through the challenges of high-bandwidth signal processing and intelligent optimization to establish a reusable flight test communication architecture. The proposed signal optimization algorithm integrates multiple indicators such as signal-to-noise ratio and spectrum, achieving dynamic optimization and selection in multi-site environments, thereby enhancing the real-time performance and stability of data transmission.

## 2. Related work

Dynamic optimization of communication transmission links is a key technology for ensuring the efficient transmission of telemetry data. Traditional methods primarily rely on channel state information and signal strength for link selection, such as the Minimum Mean Square Error (MMSE) algorithm [1] and the Maximal-Ratio Combining (MRC) algorithm [2]. However, these methods often perform poorly in complex and variable communication environments [3,4]. In recent years, with the development of machine learning technology, dynamic link selection models based on deep reinforcement learning have gradually emerged, capable of adaptively optimizing transmission paths and improving transmission efficiency. For example, link optimization algorithms based on fuzzy logic and link selection methods combining game theory and genetic algorithms have, to some extent, enhanced the robustness and performance of link selection [5,6]. Nevertheless, existing algorithms still suffer from high computational complexity and insufficient real-time performance in high-bandwidth scenarios, and link switching delays in dynamic environments remain a pressing issue.

The multi-receiver link mechanism enhances data transmission reliability through parallel reception and is a significant area of current research. Studies mainly focus on diversity reception and cooperative reception technologies [7,8], as well as load balancing and fault tolerance mechanisms. The introduction of technologies such as intelligent reflecting surface-assisted millimeter-wave communication schemes and multi-receiver cooperative frameworks [9,10] has significantly improved signal quality and transmission efficiency. However, resource allocation and interference suppression in multi-receiver links remain challenging. For instance, while dynamic load balancing algorithms and redundant receiving node mechanisms have improved link utilization and system fault tolerance [11,12], the multi-receiver synchronization problem in high-bandwidth scenarios has not been fully resolved.

Bit synchronization and frame synchronization are crucial steps in telemetry signal parsing, essential for ensuring the accuracy and reliability of data transmission. Bit synchronization ensures that the receiving end can correctly decode each data bit by accurately extracting the symbol clock information [13], while frame synchronization is responsible for identifying the starting position and format of data frames [14]. Current research primarily focuses on designing efficient synchronization algorithms for complex channel environments. In bit synchronization, algorithms based on maximum likelihood estimation optimize clock phase to improve accuracy under low signal-to-noise ratio conditions [15], and wavelet transform-based methods leverage time-frequency analysis to suppress multipath interference [16–19]. regarding frame synchronization, detection algorithms optimized with preamble correlation enhance identification capabilities [20,21], and methods combined with Kalman filtering improve robustness in time-varying channels by tracking signal parameter changes [22–26].

Despite these improvements, synchronization stability in highly dynamic environments remains a major challenge. In scenarios with high-speed motion or strong interference, signals may experience severe Doppler shifts, rapid fading, and phase jitter, causing a sharp decline in the performance of traditional synchronization algorithms. Furthermore, as communication bandwidth continues to increase, the adaptability of existing methods to high-bandwidth signals needs verification. Especially in ultra-wideband or millimeter-wave communication systems, balancing synchronization accuracy with computational complexity requires further exploration.

## 3. Models and methods

### 3.1 Simulation model

**Fig 1:**
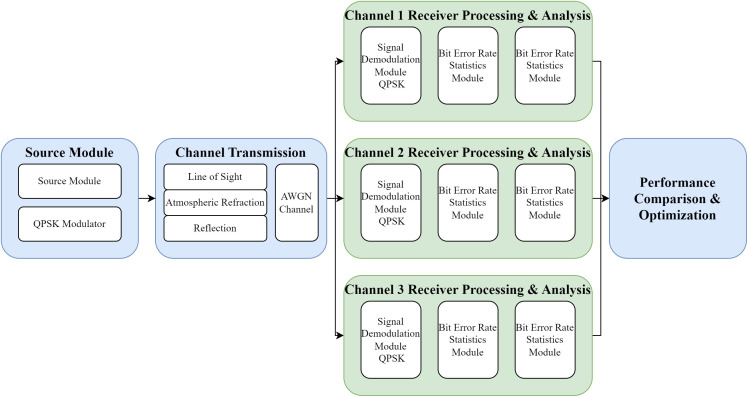
Simulation model design.

This study constructs a simulation model for a telemetry receiving system, capable of comprehensive simulation and performance evaluation for various typical transmission channels, including line-of-sight, atmospheric refraction, and reflection channels. The simulation model adopts a modular design, consisting of five main functional modules: a signal modulation module, a channel transmission module, a signal demodulation module, a bit error rate statistics module, and a constellation diagram analysis module. Its system architecture is shown in Fig 1.

In the signal processing flow, the source module generates a random binary data stream as the test signal source, simulating the digital signal transmitted in an actual telemetry link, which serves as the input for the subsequent modulator. The modulator performs phase encoding on the input binary data, mapping it to QPSK constellation points, where every two bits correspond to one symbol, distinguished by different phase states. The AWGN channel is used to simulate thermal noise or other Gaussian-like noise interference that the signal encounters in a real environment. By setting different signal-to-noise ratios, simulations are conducted for various channel fading levels and different stages to observe system performance.

In the receiver-side processing stage, the receiver’s QPSK demodulation performs coherent or non-coherent demodulation on the noisy signal to recover the original digital bitstream. Its demodulation performance directly affects the final bit error rate. The bit error rate calculation module compares the bitstream output by the demodulator with the originally transmitted bitstream, counting the cumulative number of bit errors over a given transmission length or time to calculate the actual signal-to-noise ratio. The bit error rate calculation for different branches corresponds to different simulation parameters. The constellation diagram analysis module visually displays the distribution characteristics of the received signal’s constellation points, allowing for the analysis of the impact of factors such as noise interference, phase offset, and gain imbalance on signal quality.

In multi-receiver simulation scenarios, the system supports parallel testing with different noise environment parameters. Each receiving channel independently completes signal demodulation, bit error rate calculation, and constellation diagram analysis. Finally, the optimal parameter configuration is automatically selected through a comprehensive performance comparison. This design enables the model to fully evaluate system performance under different channel conditions, modulation parameters, and filter configurations, providing data support for parameter optimization in practical engineering applications.

**Fig 2:**
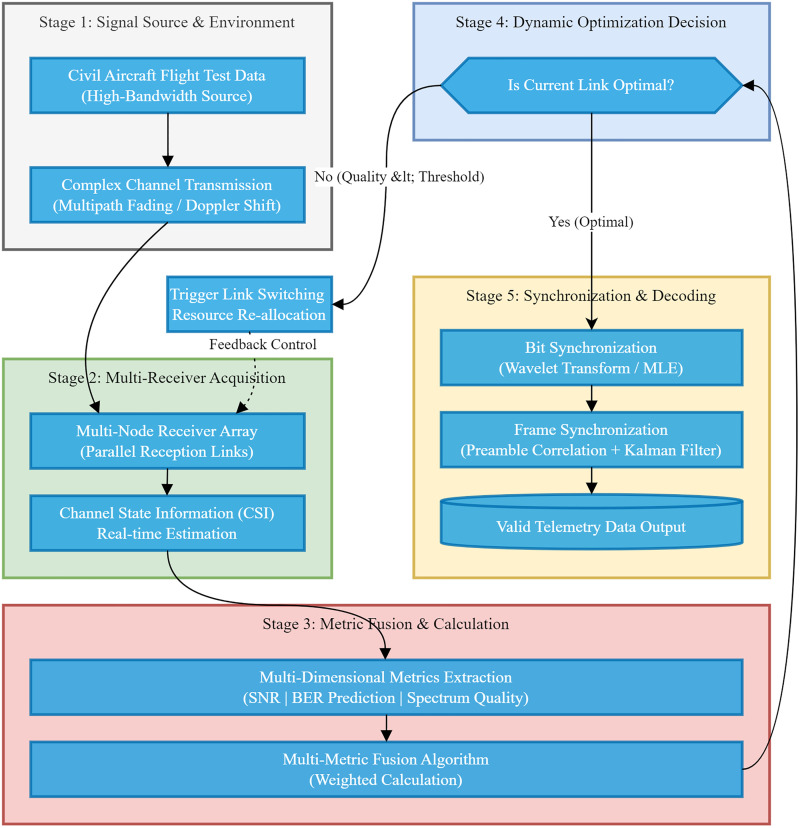
System flowchart.

The proposed dynamic optimization system, as illustrated in Fig 2, operates through a hierarchical, top-down architecture designed to ensure robust telemetry data transmission under complex channel conditions. The process begins with the acquisition of high-bandwidth signals by a multi-node receiver array, where the system contends with challenges such as multipath fading and Doppler shifts. To accurately perceive the time-varying environment, the system performs real-time CSI estimation and extracts multi-dimensional metrics, including SNR, predicted BER, and spectrum quality. These heterogeneous indicators are then synthesized by a Multi-Metric Fusion Algorithm to generate a comprehensive link quality score. This score drives the core Dynamic Optimization Decision module, which acts as the system’s control center; if the current link quality falls below an optimal threshold, a feedback mechanism triggers an adaptive link switching or resource re-allocation strategy. Conversely, when an optimal link is established, the signal undergoes advanced processing to guarantee the final output of valid and reliable telemetry data.

### 3.2 Optimal communication transmission link algorithm

The optimization problem is defined as identifying a path π from a source node s to a destination node t within the wireless self-organizing network graph. The objective is to minimize the multi-metric weighted cost while simultaneously satisfying constraints on path reliability and the maximum hop count. Notably, the hop count limit L is progressively relaxed through the outer layer of the Expanding Ring Search mechanism. The mathematical formulation of this problem is presented in Equation 1.


$$\begin{array}{c} \begin{array}{c} @l\begin{array}{cc} \underset{\pi \in \text{P}(s,t)}{\text{min}}\; & W(\pi )=\sum_{(i,j)\in \pi }\left[\alpha \;\Phi_{E}\left(d_{ij}^{\gamma }\right)+\beta \;\Phi_{D}\left(d_{ij}\right)+c_{\text{rel}}\;\Phi_{R}\left(-\text{log}p_{ij}\right)\right]\\ \text{s}.\text{t}.\; & \prod_{(i,j)\in \pi }p_{ij}\;\geq \;R_{\text{min}},\\ & |\pi |-\text{1}\;\leq \;L,\\ & (i,j)\in \pi ⟹d_{ij}\leq R_{\text{comm}},\\ & p_{ij}=\left\{ \begin{array}{cc} \;\;\;\;\;\;\;\;\;\;\;\;\;\;\;\;\;\;\;\text{max}\left\{\text{exp}\left(-{\left(\frac{d_{ij}}{d_{\text{0}}}\right)}^{\eta }\right),\;p_{\text{min}}\right\}, & (\text{Distance}\;\text{Model})\\ \text{max}\left\{(\text{1}-Q\left(\sqrt{\text{2}\;{\text{SNR}}_{ij}}\right){)}^{L_{\text{bits}}},\;p_{\text{min}}\right\}, & (\text{SNR}/\text{BER }\text{M}\text{odel}) \end{array}\right.\;\\ & \text{ERS}:\;L\;-\;L_{\text{0}},L_{\text{0}}+\Delta L,\ldots ,L_{\text{max}}\;\text{Increase}\;\text{in}\;\text{sequence}\;\text{until}\;\text{the}\;\text{first}\;\text{feasible}. \end{array} \end{array} \end{array}$$ (1)

Where $\Phi_{E},\Phi_{D},\Phi_{R}$ are operators that normalize and standardize the set of edges in the entire graph; P(s,t) is the set of all simple paths from s to t. If this is infeasible under all $L\leq L_{\text{max}}$, finding the $L_{\left(free-mini\right)}$ path to the target and use it as the best-effort. The entire algorithm consists of 5 parts.

(1) Network and Topological Model

Consider a wireless ad hoc network defined on a finite node set V={1,…,N} with designated source and destination s,t∈V,s≠t. Each node i is associated with planar coordinates $\left(x_{i},y_{i}\right)\in R^{\text{2}}$. A candidate route is represented by a simple path $\pi =\left(v_{\text{0}},\ldots ,v_{K}\right)$ from $v_{\text{0}}=s$ to $v_{K}=t$ with hop count K=|π|−1. The collection P(s,t) of all such simple paths grows exponentially in dense graphs, rendering explicit enumeration computationally infeasible and motivating algorithmic formulations based on constrained shortest-path methods, multi-label dynamic programming, or integer programming. Communication connectivity is modeled through a RGG: an undirected edge (i,j) exists if and only if the Euclidean distance $d_{ij}=\sqrt{{\left(x_{i}-x_{j}\right)}^{\text{2}}+{\left(y_{i}-y_{j}\right)}^{\text{2}}}$ satisfies $d_{ij}\leq R_{\text{comm}}$. Under a spatially uniform node distribution over area A, the expected mean degree is approximated by $\bar{k}\approx (N-\text{1})\pi R_{\text{comm}}^{\text{2}}/A$, while a classical asymptotic connectivity threshold radius satisfies $R_{c}\approx \sqrt{\left(\text{log}N)/(\pi N\right)}\sqrt{A}$; in practical deployments this threshold is typically inflated by 10–20% to enhance robustness against stochastic fluctuations and boundary effects. Since edge costs are strictly positive, any cost-minimizing path is inherently simple, eliminating the need for explicit cycle-elimination constraints.

(2) Link Reliability and Primitive Cost Components

The per-link success probability $p_{ij}$ constitutes the probabilistic substrate for reliability-constrained routing. Two modeling paradigms are considered. A distance-based exponential attenuation model $p_{ij}=\text{exp}\left(-{\left(d_{ij}/d_{\text{0}}\right)}^{\eta }\right)$ with scale $d_{\text{0}}$ and path-loss exponent η∈[2,4] affords analytical simplicity and monotonicity but omits shadowing, fast fading, and interference coupling. A physical-layer cascade comprising large-scale path loss $PL_{ij}=PL_{\text{0}}+\text{1}\text{0}n{\text{log}}_{\text{1}\text{0}}\left(d_{ij}/d_{\text{0}}^{\text{ref}}\right)$, received power $P_{r,ij}^{\text{dBm}}=P_{t}^{\text{dBm}}-PL_{ij}$, signal-to-noise ratio ${\text{SNR}}_{ij}^{\text{dB}}=P_{r,ij}^{\text{dBm}}-N_{\text{0}}^{\text{dBm}}$ with linear form ${\text{SNR}}_{ij}={\text{1}\text{0}}^{{\text{SNR}}_{ij}^{\text{dB}}/\text{1}\text{0}}$, bit error rate for BPSK $P_{b}=Q\left(\sqrt{\text{2}{\text{SNR}}_{ij}}\right)$. To ensure numerical stability in subsequent logarithmic transforms, a lower truncation $p_{ij}=\text{max}\{p_{ij},p_{\text{min}}\}$ is enforced, avoiding divergence of $-\text{log}p_{ij}$ and mitigating floating-point underflow in reliability products. The standard assumption of statistical independence across links facilitates multiplicative path reliability but may lead to optimistic estimates under correlated shadowing or interference; conservative adjustments can partially compensate. Three primitive raw edge cost components are defined: energy $E_{ij}^{\text{raw}}=d_{ij}^{\gamma }$ with γ=2 for free-space propagation and γ>2 for obstructed or multipath environments, latency surrogate $D_{ij}^{\text{raw}}=d_{ij}$, and reliability penalty $R_{ij}^{\text{raw}}=-\text{log}p_{ij}$ which exhibits local linearity in $\text{1}-p_{ij}$ near $p_{ij}=\text{1}$ and convex growth penalizing low-quality links. Empirical collinearity among these metrics motivates normalization and balanced weighting.

(3) Normalization and Composite Edge Weighting

To reconcile heterogeneous physical units and confer interpretability upon weighting coefficients, each raw cost matrix $X^{\text{raw}}\in \{E^{\text{raw}},D^{\text{raw}},R^{\text{raw}}\}$ is subjected to a global monotonic normalization mapping $\Phi_{X}$, producing dimensionless quantities ${\hat{X}}_{ij}$. A conventional Min–Max transform ${\hat{X}}_{ij}=\left(X_{ij}^{\text{raw}}-X_{\text{min}}\right)/\left(X_{\text{max}}-X_{\text{min}}\right)$ is adopted with degeneracy safeguards: if $X_{\text{max}}-X_{\text{min}}<\varepsilon$, a constant value $c_{\text{deg}}$ is assigned to avert numerical instability. Subsequent aggregation employs a linear scalarization $w_{ij}=\alpha {\hat{E}}_{ij}+\beta {\hat{D}}_{ij}+c_{\text{rel}}{\hat{R}}_{ij}$ with nonnegative coefficients $\alpha >\text{0},\beta \geq \text{0},c_{\text{rel}}\geq \text{0}$. Larger α emphasizes energy efficiency, larger β prioritizes lower geometric path length and hop count, and larger $c_{\text{rel}}$ enforces stricter rejection of unreliable links.

(4) Path Aggregation, Constraints, and Solution Methodologies

Given composite weights, the end-to-end path cost is $W(\pi )=\sum_{(i,j)\in \pi }w_{ij}$, and, under independence, the end-to-end reliability is $P(\pi )=\prod_{(i,j)\in \pi }p_{ij}$. Logarithmic transformation produces the additive risk accumulation $-\text{log}P(\pi )=\sum_{(i,j)\in \pi }\left(-\text{log}p_{ij}\right)$, so that the feasibility constraint $P(\pi )\geq R_{\text{min}}$ is equivalently expressed as a linear resource bound $\sum_{(i,j)\in \pi }\left(-\text{log}p_{ij}\right)\leq -\text{log}R_{\text{min}}$. Imposition of an additional hop cardinality limit |π|−1≤L yields a bi-resource constrained shortest path instance, known to be NP-hard for two or more additive constraints. A mixed-integer (binary) programming formulation proceeds by orienting edges to form arc set $\vec{E}$ and introducing variables $x_{ij}\in \{\text{0},\text{1}\}$ indicating arc selection. The model minimizes $\sum_{(i,j)\in \vec{E}}w_{ij}x_{ij}$ subject to flow conservation constraints $\sum_{j}x_{ij}-\sum_{j}x_{ji}=\text{1}$ for i=s, −1 for i=t, and 0 otherwise, a risk budget constraint $\sum_{(i,j)\in \vec{E}}\left(-\text{log}p_{ij}\right)x_{ij}\leq -\text{log}R_{\text{min}}$, and a hop bound $\sum_{(i,j)\in \vec{E}}x_{ij}\leq L$.

(5) Expanding Ring Search Framework and Integrated Methodological Perspective

An expanding ring search framework orchestrates progressive relaxation of the hop constraint by iterating over a sequence $L=L_{\text{0}},L_{\text{0}}+\Delta L,\ldots ,L_{\text{max}}$. For each hop budget L, an inner constrained solver is invoked to solve the bi-resource constrained subproblem. The earliest path satisfying the reliability requirement $P(\pi )\geq R_{\text{min}}$ is accepted, thereby operationalizing a minimal-hop-feasible selection principle. If no feasible path is identified up to $L_{\text{max}}$, the hop constraint is removed and a global minimum-W(π) path is produced as a best-effort solution, together with explicit reporting of its attained reliability relative to $R_{\text{min}}$. Prior to invoking the inner solver at a given L, an optimistic reliability upper bound can be computed; if this bound falls below $R_{\text{min}}$, the iteration is skipped, thereby accelerating convergence.

### 3.3 Bit synchronization signal optimization

The multi-reception optimization module employs a multi-dimensional signal optimization method based on SNR, spectral analysis, and eye diagram analysis, achieving efficient screening and quality assessment of received signals. First, the module calculates the SNR of each received signal in real-time using noise power spectral density estimation and RMS power calculation methods, as shown in Equations 2–5, and quickly screens for candidate signals that meet preset thresholds. Subsequently, the system performs a FFT on the candidate signals to generate corresponding spectrograms and uses image processing algorithms for in-depth analysis of spectral characteristics. During this process, the system can effectively identify and eliminate signals with abnormal characteristics such as spectral leakage and interference clutter, thereby ensuring the spectral quality of the selected signal. This multi-dimensional optimization method enhances the accuracy and reliability of signal screening, providing high-quality input for subsequent signal processing.


$$\begin{array}{c} SNR=\text{1}\text{0}{\text{log}}_{\text{1}\text{0}}\left(\frac{P_{signal}}{P_{noise}}\right) \end{array}$$ (2)

$$\begin{array}{c} \frac{Eb}{N\text{0}}=\text{1}\text{0}{\text{log}}_{\text{1}\text{0}}\left(\frac{P_{signal}}{R\cdot P_{noise}}\right) \end{array}$$ (3)

$$P_{signal}=\;\frac{\text{1}}{N}\sum_{i=\text{1}}^{N}x(i{)}^{\text{2}}$$ (4)

$$\begin{array}{c} P_{noise}=\frac{\text{1}}{B}\int_{f_{\text{1}}}^{f_{\text{2}}}S_{noise}(f)df \end{array}$$ (5)

In the formula, *x*(*i*) represents the signal sample value; *N* is the number of sampling points, and *R* is the code rate; *S*_*noise*_ represents the noise power spectral density; *f* is the communication frequency.

**Fig 3:**
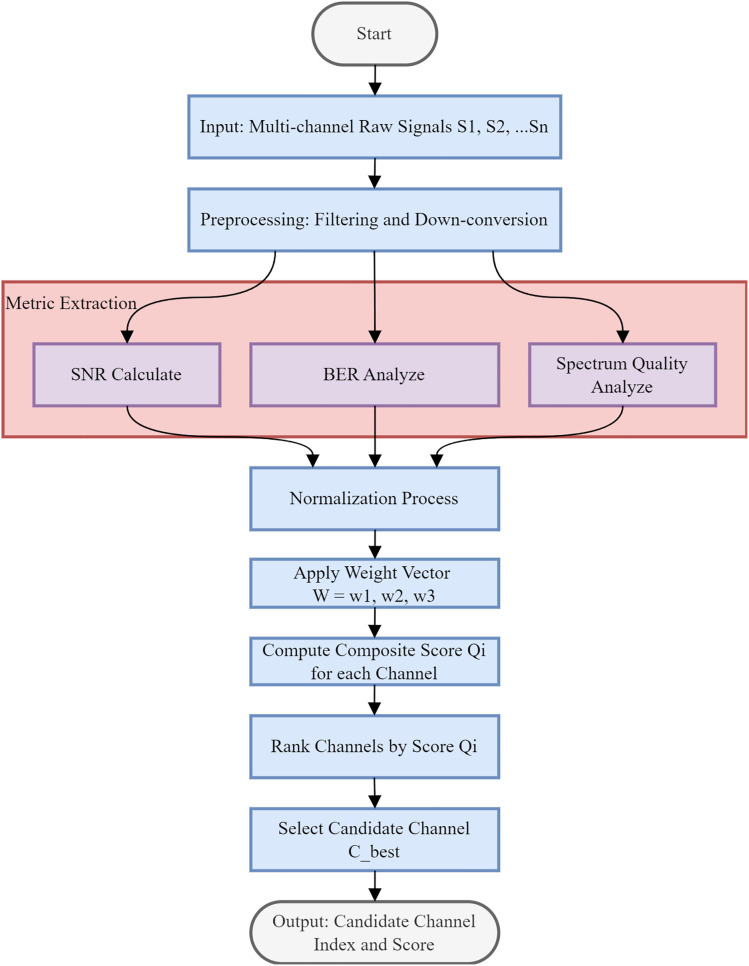
Multi-dimensional signal optimization algorithm.

The proposed methodology delineates a systematic framework for optimal channel selection in multi-channel communication systems, designed to maximize link reliability and spectral efficiency. As illustrated in Fig 3, the algorithm accepts a set of multi-channel raw signals ($S_{\text{1}},S_{\text{2}},\ldots ,S_{n}$) as input. These signals first undergo a rigorous preprocessing phase, which incorporates filtering and down-conversion techniques to suppress out-of-band interference and translate the carrier frequency to a baseband or intermediate frequency suitable for analysis. Central to the assessment strategy is the Metric Extraction block, which performs a parallel evaluation of three critical performance parameters: SNR, BER, and Spectrum Quality. By simultaneously analyzing these distinct characteristics, the system captures a holistic view of the physical layer conditions, effectively balancing signal strength, transmission accuracy, and spectral purity.

Following feature extraction, the algorithm employs a weighted decision matrix to identify the superior channel. Since the extracted metrics possess varying units and scales, a normalization process is executed to map the data onto a uniform dimensionless range. A weight vector $W=\left[w_{\text{1}},w_{\text{2}},w_{\text{3}}\right]$ is subsequently applied, providing the flexibility to adjust the significance of SNR, BER, or spectrum quality according to specific QoS constraints. A composite quality score, denoted as $Q_{i}$, is calculated for each channel via a linear combination of these weighted normalized values. The channels are then ranked in descending order based on their respective $Q_{i}$ values. The process culminates in the selection of the candidate channel $C_{best}$, outputting both its index and calculated score to facilitate the final link establishment. The average channel switching delay is controlled within 5 ms. The hysteresis mechanism effectively prevents frequent switching, thereby reducing the total synchronization overhead compared to traditional threshold-based methods.

### 3.4 Frame synchronization signal optimization

Utilizing data-driven optimization techniques, intelligent optimization is performed on the four-channel transmission data, including two methods: bit voting and locked weighted voting. The bit voting method achieves data optimization through data alignment and bit-by-bit counting, suitable for application scenarios with three or more channels. The locked weighted voting algorithm enhances the system’s robustness in high-interference environments by dynamically evaluating channel reliability.

The core of the locked weighted algorithm lies in the quantitative evaluation of the channel’s synchronization status, primarily examining three key indicators: lock duration, lock stability, and bit error distribution. Among these, lock duration reflects the continuous time a channel maintains stable synchronization; lock stability indicates whether the channel has frequently experienced loss of lock in a recent period or a specified number of data frames; bit error distribution is used to measure the bit error rate of the channel during the locked phase, assisting in judging the severity of its fading or multipath interference.

The specific implementation includes four key steps: First, perform time-level or frame-level alignment of multi-channel data to eliminate clock deviations and frame start differences; Second, continuously monitor the carrier synchronization, bit synchronization, and frame synchronization status of each channel; Third, dynamically assign weights to each channel based on the weighting function shown in Equation 6 and the synchronization status evaluation results, with each channel being allocated a corresponding weight in the fusion decision. Channels that are more stable, have a longer lock duration, and a lower recent loss-of-lock rate receive higher weights; Fourth, use a sliding window mechanism for iterative weight updates. This algorithm supports multiple weighting strategies, including linear weighting and exponential decay weighting, which can be flexibly selected according to the actual application scenario.


$$\begin{array}{c} w_{i}=f\left({LockDuration}_{i},\;{LockStability}_{i},{ErrorRate}_{i}\right) \end{array}$$ (6)

The weighted sum of the received bit values or modulation symbols from each channel and their corresponding weights is used as the decision reference benchmark, optimizing the process for the bit or symbol at each decision instant. In binary digital communication, a binary decision is made on the weighted result based on a threshold; for multi-level modulation systems, the optimal symbol decision is made using the minimum Euclidean distance criterion or the maximum likelihood estimation method based on Equation 7. This layered decision mechanism ensures decision efficiency in binary communication and guarantees decision accuracy in higher-order modulation systems, allowing the algorithm to adapt to communication scenarios of varying complexity. The introduction of the weighted sum effectively integrates the reliability information from each channel, while the selection of an adaptive decision criterion enhances the system’s performance under different modulation schemes.


$$\begin{array}{c} b_{out}=decide\sum_{i}w_{i}b_{i} \end{array}$$ (7)

The locking weighted algorithm employs a dynamic weight adjustment mechanism. By monitoring the locking status and demodulation performance of each channel in real-time, it quantifies channel reliability into weighting coefficients, which are updated after each data fusion based on decision results and bit error situations. This mechanism can effectively adapt to fluctuations in channel quality caused by environmental changes. The main features of this algorithm include: (1) a rapid weight response mechanism that can quickly reduce the impact of a channel when it experiences interference or loss of lock; (2) dynamic adaptability to handle complex scenarios such as aircraft maneuvers and terrain obstruction; (3) automatic optimization of the combining strategy by analyzing the demodulation performance of each channel. In multi-receiver scenarios like civil aircraft telemetry and high-speed communications, this algorithm significantly improves system stability, reduces the bit error rate, and enhances robustness, with its advantages being particularly prominent in complex interference environments.

**Fig 4:**
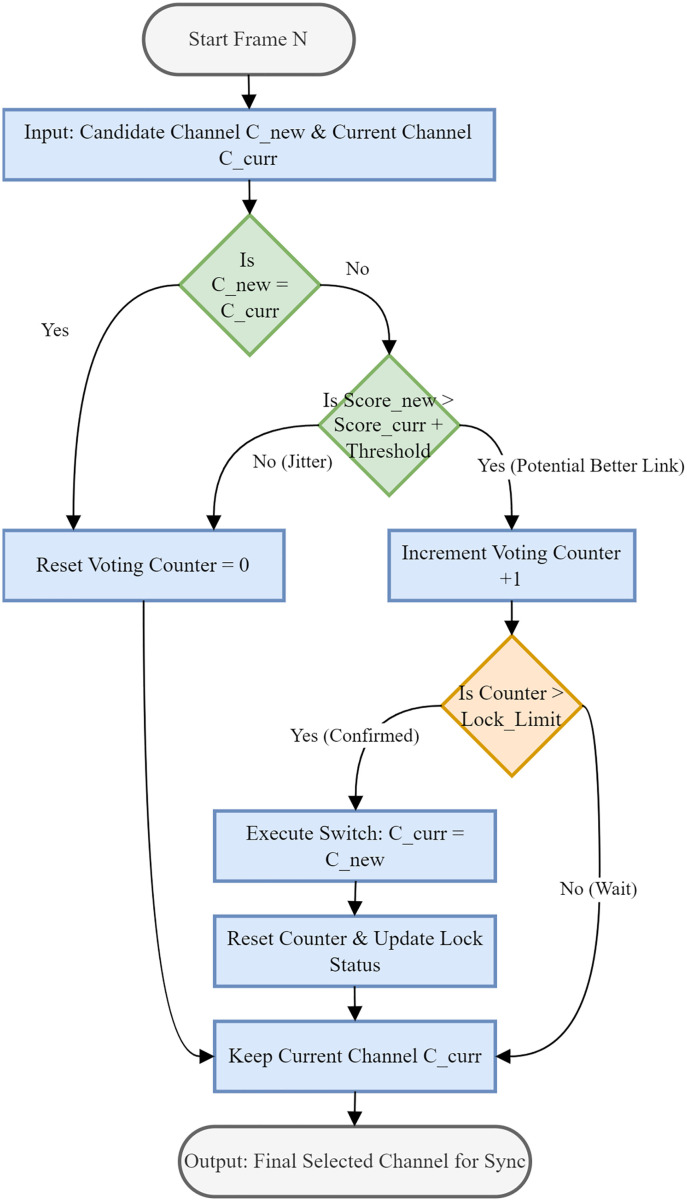
Locked weighted voting algorithm.

To guarantee the continuity of the data link and eliminate the instability associated with the “ping-pong” effect—where the system oscillates rapidly between channels of comparable quality—a robust, hysteresis-based decision logic is employed for the final channel selection, as shown in Fig 4. This stage operates immediately after the metric-based ranking process, taking the optimal candidate channel ($C_{new}$) and the currently locked channel ($C_{curr}$) as inputs for the N-th frame. Rather than adopting a greedy approach that instantaneously switches to the highest-scoring channel, the proposed algorithm implements a dual-layer filtering mechanism comprising both amplitude hysteresis and temporal persistence verification to validate the necessity of a handover.

Initially, the algorithm verifies whether the candidate channel differs from the current one; if they are identical, the system recognizes the stability of the current link, resets the voting counter to zero, and maintains the status quo. However, if a different candidate is proposed, the algorithm evaluates the magnitude of the performance gain. A switch request is validated only if the composite score of the new channel exceeds that of the current channel by a pre-configured margin, denoted as the Threshold ($Score_{new}>Score_{curr}+Threshold$). This amplitude hysteresis serves as a primary noise filter, ensuring that minor spectral variations or measurement jitter do not trigger unnecessary handovers that could disrupt frame synchronization. Furthermore, to prevent switching due to transient, impulse-like interference that might cause a momentary spike in a non-active channel’s score, a temporal voting mechanism is engaged. A voting counter increments for each consecutive frame where the threshold condition is met. If at any point the condition fails—indicating the signal improvement was sporadic—the counter is reset, preventing premature transitions. The actual execution of the channel switch, where $C_{curr}$ is updated to $C_{new}$, is strictly conditional upon this counter exceeding a specific Lock_Limit. This requirement ensures that the superior performance of the candidate channel is not an anomaly but a sustained trend confirmed over a defined time window. By integrating these amplitude and time-domain constraints, the algorithm effectively suppresses frequent re-synchronization events, thereby significantly enhancing the overall robustness of the telemetry system and ensuring reliable data demodulation even in highly dynamic signal environments where signal fading and recovery are common.

## 4. Results and discussion

### 4.1 Communication link networking and optimization

**Fig 5:**
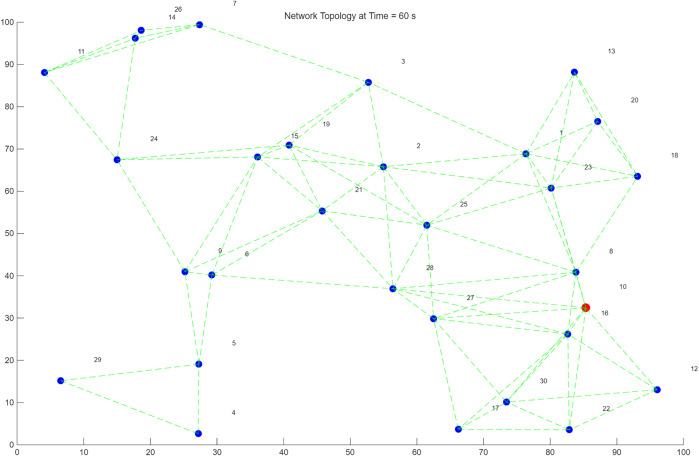
High-speed source mobility simulation results.

**Fig 6:**
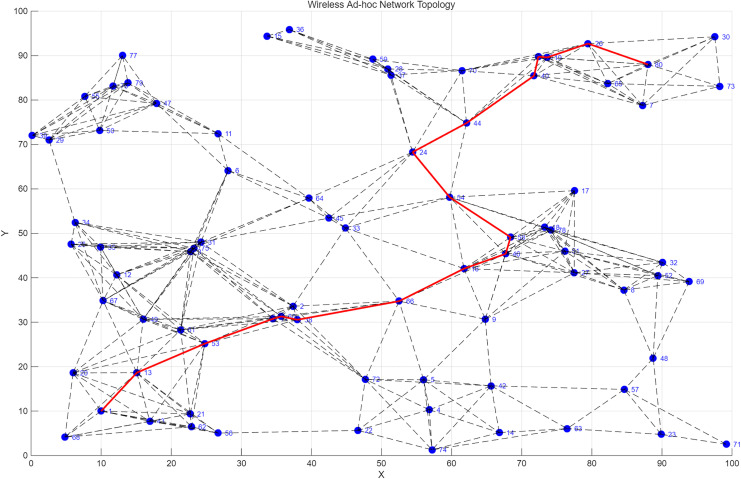
Low-speed source mobility simulation environment.

In a wireless ad hoc network, the network topology and connection relationships at a specific moment, based on a multi-reception link mechanism. Figs 5 and 6 illustrates the networking process during high-speed and low-speed movement of the source node, respectively. Nodes are represented by dots, and each node has dynamic coordinate attributes in the simulation environment. The network topology changes dynamically over time to maintain the stability of link communication. The connections between nodes are represented by two types of lines: dashed lines represent effective communication links detected at the current moment, which change dynamically due to factors like node movement and channel fading; solid lines represent the transmission path determined by the optimal path selection algorithm, which achieves an optimal state in terms of both data transmission rate and stability. The multi-reception link mechanism enables the network to dynamically select multiple feasible transmission paths, effectively avoiding single-path congestion and link interruption issues. Simulation results show that this mechanism’s performance improvement is particularly significant in network scenarios with high node density or frequent topological changes, furthermore, the optimized routing strategy minimizes the hop count, maintaining the end-to-end transmission latency at a low level suitable for real-time telemetry monitoring.

Compared to the traditional AODV protocol, the multi-receive link mechanism has significant advantages. As can be seen from Table 1, the improved LEACH variants generally perform better in terms of energy consumption, network lifetime, and energy balancing, while the improved AODV variants have an edge in adapting to highly dynamic networks and scalability. Under the multi-receive link mechanism, a node can simultaneously receive and forward routing information from multiple neighboring nodes, a feature that significantly enhances the robustness of the ground test network. When a certain route fails due to node movement or deteriorating link quality, the node can quickly switch to other recorded backup paths, thereby effectively reducing network downtime and increasing the data transmission success rate.

**Table 1:** Comprehensive performance comparison of various improved algorithms.

Algorithm	Energy Consumption	Network Lifetime	Routing Overhead	Adaptability (Highly Dynamic Networks)	Adaptability (Static Networks)	Data Aggregation Capability	Scalability Implementation	Complexity	Energy Balancing	Cluster Head Selection Optimization
Standard AODV	High	Short	High	High	Medium	No	Medium	Low	Low	N/A
G-AODV	MediumLow	MediumLong	Medium	MediumHigh	Medium	No	MediumHigh	Medium	Medium	N/A
AODV-ECA	Medium	MediumLong	MediumHigh	High	Medium	No	Medium	MediumHigh	High	N/A
RSSI-AODV	Medium	Medium	Medium	High	Medium	No	Medium	Medium	Medium	N/A
LEACH	Medium	Medium	Medium	Low	High	Yes	Low	Low	Medium	Low
AE-LEACH	Low	Long	Low	Medium	High	Yes	Medium	MediumHigh	High	High
ERA-EACH	Low	Long	Low	Medium	High	Yes	Medium	Medium	High	High
MFG-EACH	MediumLow	Long	Medium	MediumHigh	High	Yes	MediumHigh	High	High	High
LEACH-M	Medium	MediumLong	Medium	MediumHigh	MediumHigh	Yes	Medium	MediumHigh	Medium	Medium

### 4.2 Optimal selection of multiple received signals

#### 4.2.1 Optimal selection of bit synchronization signals.

**Fig 7:**
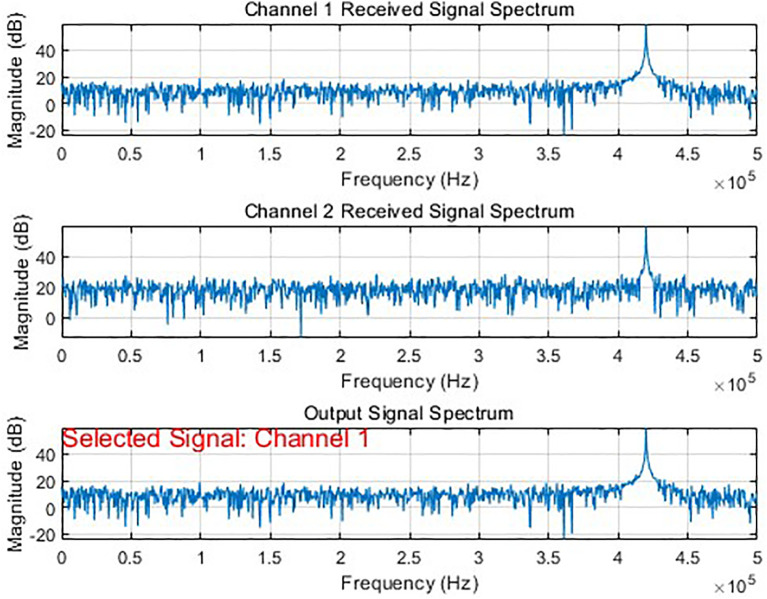
Optimization results of bit synchronization signal (channel 1 selected).

**Fig 8:**
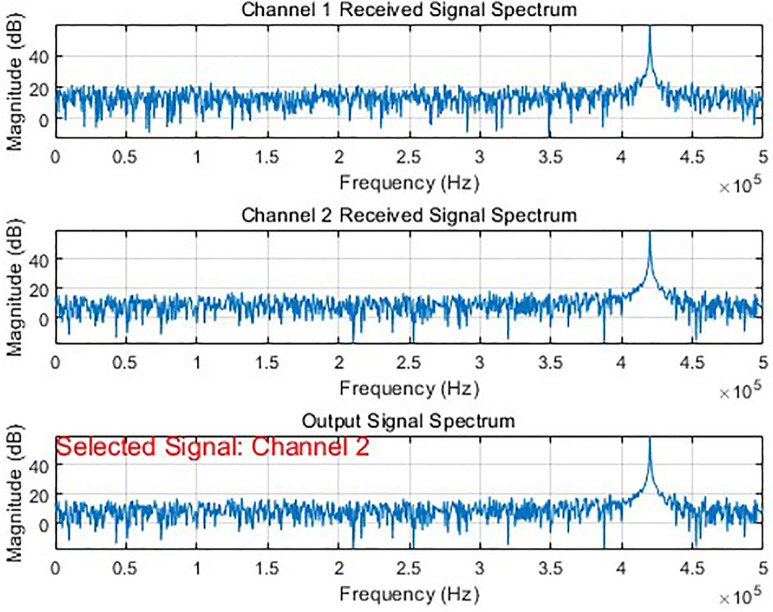
Optimization results of bit synchronization signal (channel 1 selected).

In simulation experiments with signals of different parameter configurations, by dynamically adjusting parameters such as the transmission power, channel gain, and fading of the communication link, the system can accurately identify the optimal communication link and achieve adaptive switching. As shown in Figs 7 and 8, the system can correctly select and output the source signal from the best channel under different simulation scenarios.

During the simulation, by dynamically adjusting key parameters such as transmission power, channel fading factors, and gain coefficients, we effectively modeled the channel state variations caused by changes in distance, obstacle obstruction, and multipath effects in actual wireless transmission. The experimental results show that when the system detects that other channels exhibit superior performance in a changing transmission environment such as an improved signal-to-noise ratio, it can switch from the current channel to a better one in real time.

By perceiving the signal quality indicators of each link in real time, this algorithm implements an adaptive communication scheduling and dynamic switching mechanism. This rapid switching capability among multiple channels significantly enhances the system’s transmission reliability, effectively overcoming interference and fading issues in wireless communication, and providing a viable solution for stable communication in complex wireless environments.

#### 4.2.2 Frame synchronization signal optimization.

**Fig 9:**
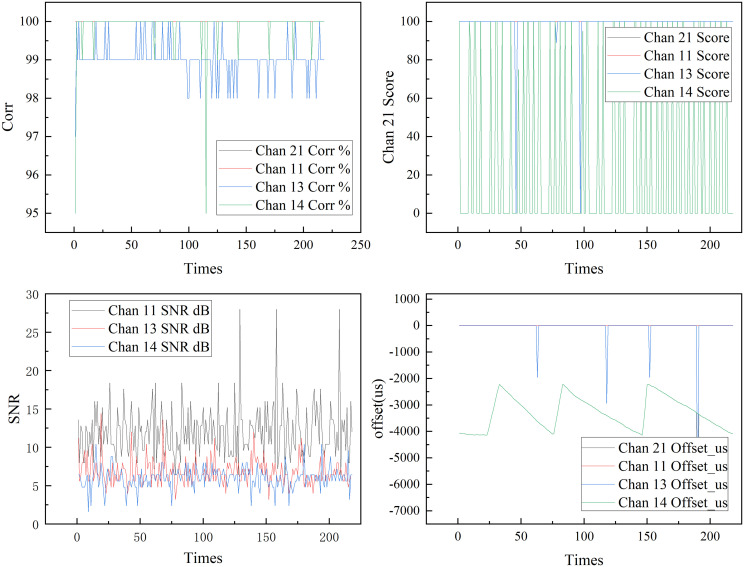
Optimization results of frame synchronization signal.

The signals from the 3 channels are optimized, and the output is through channel 21, as shown in Fig 9.

By dynamically selecting the optimal transmission channel through real-time evaluation and comparison of the performance parameters of each channel in the telemetry system. Among these, signal correlation is used to quantify the degree of match between the received signal and the reference signal. A higher value indicates a better fit between the channel signal and the target pattern, and typically, a higher decoding success rate. The channel quality score, as a comprehensive evaluation metric, works together with the SNR to reflect the channel’s transmission quality, ensuring stable signal output under different testing environments and obstruction conditions. The synchronization offset is used to monitor the channel’s synchronization status. When this value is large or fluctuates dramatically, it often indicates that the channel may be experiencing a loss of synchronization or demodulation errors. As seen in Fig 7, channel 21, as the selected output channel, corrects the synchronization offset of each channel among channels 11, 13, and 14 based on metrics like signal Corr, Score, and SNR, consistently maintaining the output of the channel with the best current quality and keeping the frame synchronization locked.

### 4.3 Telemetry performance evaluation

**Fig 10:**
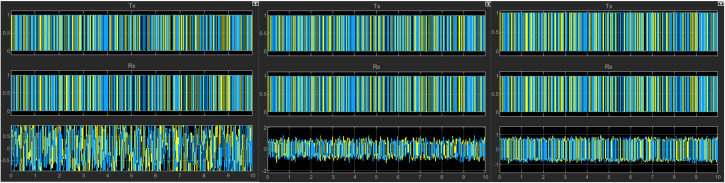
Constellations diagram.

**Fig 11:**
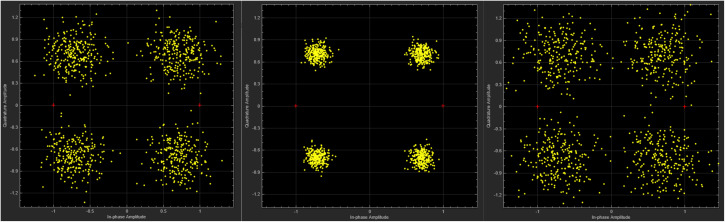
Waveform diagram.

During the simulation model’s operation, the bit error rate detection is shown in Table 2, and the constellation diagram and waveform diagram are shown in Figs 10 and 11.

**Table 2:** Output results of different bit error rate channels in channel optimization.

Bit Error Rate Analysis	T1	T2	T3	T4	T5	T6	T7	T8	T9	T10
Channel 1 Bit Error Rate	0.03922	0	0.04305	0.04478	0.0239	0.049683	0.002849	0.04115	0.04213	0.03992
Channel 1 Bit Error Count (10e3)	4	0	13	18	19	30	2	33	38	40
Channel 1 Data Volume (10e3)	102	202	302	402	502	602	702	802	902	1002
Channel 2 Bit Error Rate	0.009804	0.00901	0.009934	0.01244	0	0.01993	0.01994	0.009975	0.0122	0.01397
Channel 2 Bit Error Count (10e3)	1	2	3	5	0	12	14	8	11	14
Channel 2 Data Volume (10e3)	102	202	302	402	502	602	702	802	902	1002
Channel 3 Bit Error Rate	0	0.4455	0	0	0.005976	0.003322	0.03989	0.001247	0.002217	0.001996
Channel 3 Bit Error Count (10e3)	0	9	0	0	3	2	28	1	2	2
Channel 3 Data Volume (10e3)	102	202	302	402	502	602	702	802	902	1002
Preferred Channel	3	1	3	3	2	3	1	3	3	3

As presented in Table 2, variations in the simulation environment induce fluctuations in the BER across different receiving channels. The proposed optimal channel selection scheme dynamically identifies and locks onto the channel with the lowest BER for bit synchronization signal output. By automatically switching to a more reliable communication link during channel degradation, the system ensures data transmission reliability and efficiency. Notably, the “Preferred Channel” consistently maintains the highest data volume, guaranteeing that the effective system throughput remains stable near the theoretical maximum bandwidth, even when individual channels experience deep fading.

In the real-time monitoring and analysis of constellation diagrams and waveforms, the accuracy of automatically selecting the better performing transmission channel was verified by continuously comparing the quality scores and signal-to-noise ratio parameters of each channel. Experimental results show that dynamic signal optimization improves the decoding accuracy and transmission reliability of telemetry data, especially demonstrating good adaptability and robustness in complex electromagnetic environments. The system can promptly respond to changes in channel quality, ensuring that the optimal channel is always used for data transmission, thereby effectively improving overall communication quality.

### 4.4 Comparative analysis

To objectively evaluate the performance superiority of the proposed Locked Weighted Voting algorithm, we conducted a rigorous comparative analysis against two widely adopted traditional baseline methods: MMSE and MRC. All algorithms were evaluated under identical simulation conditions characterizing a typical high-dynamic flight test environment. The channel model utilized a Rayleigh fading channel with AWGN, simulating a flight speed of Mach 0.8 and an SNR range varying dynamically from 5 dB to 20 dB.

The BER serves as the primary metric for demodulation accuracy. As presented in Table 3, the proposed LWV algorithm demonstrates a distinct advantage, particularly in low-SNR environments. The traditional MMSE algorithm, while effective in static channels, exhibits performance degradation in highly dynamic scenarios. Its reliance on instantaneous channel estimation leads to error propagation when the channel state changes rapidly. Similarly, while MRC theoretically provides optimal SNR by coherently combining signals, its practical application in high-speed flight tests is limited. Accurate phase alignment for all branches is computationally intensive and prone to estimation errors and phase jitter caused by high-speed maneuvering often leads to constructive interference failure. In contrast, the proposed LWV algorithm achieves the lowest average BER of $\text{3}e^{-\text{6}}$. By incorporating a multi-dimensional evaluation with integrating SNR, spectral quality, and eye diagram analysis rather than relying solely on signal strength, the LWV effectively filters out channels with high power but poor signal quality, ensuring that only the most reliable data stream is selected for demodulation.

**Table 3:** Quantitative performance comparison under dynamic channel conditions.

Algorithm	BER	Frame Sync Success	Channel Switching Count	Computational Complexity
MMSE	$\text{4}.\text{2}e^{-\text{5}}$	92.5%	145	Low
MRC	$\text{1}.\text{8}e^{-\text{5}}$	95.8%	N/A	High
LWV	$\text{3}e^{-\text{6}}$	98.9%	23	Low

Finally, regarding computational feasibility, while MRC offers competitive BER performance, its complexity scales linearly with the number of receiver branches, imposing a heavy load on onboard processors. The proposed LWV algorithm employs a simplified voting logic after the initial signal quality assessment, maintaining a low computational footprint similar to MMSE. This characteristic makes it highly suitable for real-time implementation on FPGA-based telemetry hardware where processing resources are constrained. The comparative results confirm that the proposed LWV algorithm strikes an optimal balance between demodulation accuracy and system stability, outperforming traditional methods by effectively mitigating the impact of fast fading and reducing synchronization overhead.

## 5. Conclusion

Addressing the high-bandwidth, multi-receiver telemetry communication demands in civil aircraft flight tests, this study investigated a dynamic signal optimization mechanism for high-bandwidth multi-receiver points, employing methods such as channel modeling and receiver-end optimal decision-making. The following conclusions were drawn from the research:

(1) The signal optimization mechanism improved data parsing performance. Based on high-precision channel modeling and a multi-receiver dynamic optimization algorithm, it effectively solved problems such as signal occlusion, multipath effects, and noise interference, improving the demodulation efficiency and accuracy of telemetry data.
(2) The improved routing protocol optimized network performance. Through improved AODV and LEACH algorithms, efficient self-organizing routing scheduling was achieved in mobile environments, reducing energy consumption and delay, while enhancing network energy balance and reducing data loss due to node failure.
(3) The system possesses good robustness and scalability. By using a locked weighted decision algorithm, combined with an adaptive signal optimization method based on multi-dimensional indicators such as signal-to-noise ratio, spectral characteristics, and eye diagram analysis, real-time data and accuracy can still be ensured in harsh environments. Experiments verified that the proposed multi-receiver optimization method and efficient networking scheduling scheme have good robustness and scalability, suitable for conditions such as high-speed flight and complex terrain.

Future research can further explore higher-order modulation schemes and precise channel prediction models to optimize cooperative decision-making and bandwidth allocation strategies, continuously improving data transmission efficiency and system stability.
